# Pneumonia After Cardiovascular Surgery: Incidence, Risk Factors and Interventions

**DOI:** 10.3389/fcvm.2022.911878

**Published:** 2022-06-30

**Authors:** Dashuai Wang, Yang Lu, Manda Sun, Xiaofan Huang, Xinling Du, Zhouyang Jiao, Fuqiang Sun, Fei Xie

**Affiliations:** ^1^Department of Cardiovascular Surgery, The First Affiliated Hospital of Zhengzhou University, Zhengzhou, China; ^2^Department of Cardiology, The First Affiliated Hospital of Zhengzhou University, Zhengzhou, China; ^3^China Medical University-The Queen's University of Belfast Joint College, China Medical University, Shenyang, China; ^4^Department of Cardiovascular Surgery, Union Hospital, Tongji Medical College, Huazhong University of Science and Technology, Wuhan, China; ^5^Department of Vascular and Endovascular Surgery, The First Affiliated Hospital of Zhengzhou University, Zhengzhou, China

**Keywords:** cardiovascular surgery, risk factor, incidence, intervention, pneumonia

## Abstract

Postoperative pneumonia (POP) is prevalent in patients undergoing cardiovascular surgery, associated with poor clinical outcomes, prolonged hospital stay and increased medical costs. This article aims to clarify the incidence, risk factors, and interventions for POP after cardiovascular surgery. A comprehensive literature search was performed to identify previous reports involving POP after cardiovascular surgery. Current situation, predictors and preventive measures on the development of POP were collected and summarized. Many studies showed that POP was prevalent in various cardiovascular surgical types, and predictors varied in different studies, including advanced age, smoking, chronic lung disease, chronic kidney disease, cardiac surgery history, cardiac function, anemia, body mass index, diabetes mellitus, surgical types, cardiopulmonary bypass time, blood transfusion, duration of mechanical ventilation, repeated endotracheal intubation, and some other risk factors. At the same time, several targeted interventions have been widely reported to be effective to reduce the risk of POP and improve prognosis, including preoperative respiratory physiotherapy, oral care and subglottic secretion drainage. Through the review of the current status, risk factors and intervention measures, this article may play an important role in clinical prevention and treatment of POP after cardiovascular surgery.

## Introduction

With the improvement of economic conditions and living standards, the incidence of cardiovascular disease has increased year by year, with high morbidity and mortality (1). Although considerable progress have been made in diagnostic techniques, medical options and interventional therapy over the past decades, surgical operation remains one of the most important treatments (2, 3). However, the circumstances of the overall survival after cardiovascular surgery is hardly optimistic and a high proportion of patients may develop various complications (4).

Postoperative pneumonia (POP) is one of the most common pulmonary complications after cardiovascular surgery, related to poor clinical outcomes, prolonged hospital stay and increased medical costs (5, 6). The incidence of POP varies greatly in previous reports due to various definitions and surgical patients in different studies (7–10). Several studies aimed to identify predictors for the development of POP after cardiovascular surgery have been conducted and some predictors have been reported, however, those risk factors reported in different studies varied greatly (11, 12). In addition, studies focused on the prevention and treatment of POP have been carried out and several interventions have been widely reported to be effective to reduce the incidence of POP and resultant adverse outcomes (13–16). Recently, several related studies conducted in patients undergoing cardiovascular surgery have been reported, nonetheless, a comprehensive review of the current status, risk factors, and interventions for cardiovascular surgery is still lacking.

Understanding the current status and clarifying the risk factors for the development of POP may play an important guiding role for early recognition, targeted prevention and treatment. The aims of this study was to clarify the incidence, risk factors, and interventions for POP after cardiovascular surgery through comprehensive literature search, and provide new ideas for clinical prevention and treatment.

## Methods

**Figure 1:**
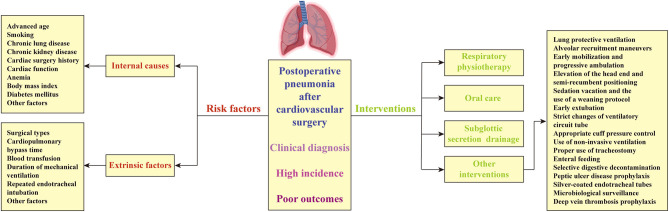
Incidence, risk factors and interventions of pneumonia after cardiovascular surgery.

We conducted a literature search of peer-reviewed publications to obtain a comprehensive review of published data on POP after cardiovascular surgery using the Cochrane, PubMed and EMBASE databases. These sources were searched up to the end of April 2022. Search terms used included a variety of synonyms and types of surgical approach, as well as pneumonia. Article titles and abstracts were screened and potentially valuable articles were read in full text. The review included only English language articles, and studies in other languages were excluded. A data extraction form was used to capture key issues in the articles. We compiled data pertaining to the number of patients, anatomical procedure sites, pneumonia rates, risk factors, preventative measures, and interventions. Primary researches, systematic reviews, studies relevant to guideline implementation and can be transferable to clinical practice were eligible for inclusion. Where appropriate the findings were also supported by additional theoretical literature. The main contents of this study have been summarized in Figure 1.

## Current Situation

POP is one of the most common pulmonary complications after cardiovascular surgery. According to the recommendation of clinical practice guidelines, the clinical diagnosis of pneumonia is mainly based on objective indicators such as clinical syndromes and chest imaging changes (17, 18). Generally speaking, POP can be diagnosed when patients have cough, sputum or sputum character changes after cardiovascular surgery, chest ray or computerized tomographic scanning shows new or progressive infiltration, consolidation or ground glass shadow, and meet two or more of the following three conditions at the same time: (1) body temperature rises to more than 38°C or falls below 36°C, (2) white blood cell count >10 × 10^9^/L or <4 × 10^9^/L, and (3) purulent secretions in the lower respiratory tract. On the basis of clinical diagnosis, etiological diagnosis can be established if one or more of the following conditions are met: (1) pathogens can be cultured in qualified lower respiratory secretions, bronchoalveolar lavage fluid or bronchoscopic anti-contamination brushes and are consistent with clinical manifestations, (2) pathogens can be seen by microscopy, cytopathology or in the lung tissues and relevant evidence of tissue damage exists, (3) history of epidemiological exposure to respiratory viruses and isolation of virus, detection of viral antigen or positive nucleic acid test.

The incidence of POP reported in previous literature varied greatly due to various surgical populations, from 2 to 15% in most of the studies (7, 9, 19, 20). The most frequently reported studies for POP were conducted in patients undergoing surgery for valvular and coronary disease, the incidence of which was mostly between 2.1–8.2% (7, 9). However, POP most frequently develops following surgery for aortic dissection, with an incidence rate of up to 36.9% in the literature (21). Although the incidence of POP varies in different studies, researchers find that the development of POP can significantly prolong the duration of intensive care unit (ICU) and hospital stay, increase the morbidity and mortality, aggravate patient's physical and mental pains, and increase their medical burden (6, 12, 22, 23).

## Risk Factors

### Internal Causes

#### Advanced Age

Advanced age has been widely reported to be associated with the development of POP after cardiovascular surgery (10, 19, 20, 24–26). In a prospective multicenter study, Ailawadi et al. found that the risk of POP after cardiac surgery increased significantly with age, presenting a dose-effect relationship (27). Kilic et al. found that compared to patients younger than 65 years, the risk of POP after cardiac surgery increased 1.4-fold in patients older than 65 years (24). For patients older than 70 years, Hortal et al. found a 3.5-fold increased risk of POP on the day of surgery, and 16.8 times for patients requiring more than 3 days of postoperative mechanical ventilation (28). In addition, advanced age has been reported to be a strong predictor of postoperative multidrug-resistant bacterial infection, closely associated with an increased risk of in-hospital mortality (29).

With the increase of patient's age, the function of various organs gradually declines, the risk of various comorbidities increases, the body's defense mechanism and immune function weaken, together resulting in a higher risk of POP after cardiovascular surgery. Compared with young patients, elderly patients have weakened cough reflex, decreased airway self-purification ability, weakened respiratory muscle strength, reduced pulmonary ventilation and air-exchanging functions, which make pathogens easier to move down the respiratory tract and then cause infection (30). In addition, the ability of elderly patients to metabolize drugs and nutrients is weakened, the ability to resist pathogenic invasion becomes poorer, the tolerance to pain, surgery, trauma and external stimuli is weakened, and the response to changes decreases, which may significantly increase the risk of POP in elderly patients. Therefore, additional attention should be given to older surgical patients to timely deal with abnormal conditions, and some targeted measures should be given in advance to prevent and reduce the risk of POP.

#### Smoking

Smoking has been identified as an independent predictor for POP after cardiovascular surgery in previous studies, which may be due to the fact that smoking can increase the risk of airway pathogens colonization (7, 19, 20). Kinlin et al. found that patients with a history of smoking had a 1.79-fold increased risk of POP after cardiac surgery, which is very close to the findings of Wang et al. (7, 19). Saxena et al. found that compared with patients who had quitted smoking or who had never smoked, current smokers had a 2.05-fold increased risk of POP after coronary artery bypass grafting (CABG) (31). Al-sarraf et al. found that compared with patients who quitted smoking for more than 4 weeks, current smokers had a significantly higher risk of pulmonary complications after CABG (32). However, compared with patients who had never smoked, the risk of POP was not significantly increased in patients who quitted for more than 4 weeks. Guidelines recommend that all smokers should receive inpatient education counseling when treated with CABG and hospitals should provide appropriate smoking cessation strategies for these patients (33). Active preoperative smoking cessation counseling and treatment can not only reduce the risk of postoperative respiratory complications, but also have a certain protective effect on the long-term postoperative survival. It has been reported that patients who quitted smoking for more than a week before surgery were 10 times more likely to remain smoking cessation within 1 year after CABG and had a significantly improved 5-year survival rate postoperatively (34, 35). Therefore, in clinical practice, not only high-risk patients, but also the general public should be encouraged to quit smoking to pursue long-term primary prevention and public health protection (36).

#### Chronic Lung Disease

Chronic lung disease is another important predictor for POP after cardiovascular surgery, which has been extensively reported in various literature (10, 24, 25). Both Kilic et al. and Strobel et al. found that the risk of POP after cardiac surgery increased significantly with increasing severity of chronic lung disease (10, 24). The findings of Wang et al. showed that patients with chronic obstructive pulmonary disease had a 2.1-fold increased risk of POP compared with patients with good respiratory conditions (19). A systematic review and meta-analysis conducted by Furukawa et al. found that preoperative inspiratory muscle training in adult patients undergoing cardiac surgery can significantly reduce the risk of POP and shorten the length of hospital stay (37). Therefore, preoperative respiratory optimization with respiratory physiotherapy may be a good option for patients undergoing elective cardiac surgery.

#### Chronic Kidney Disease

Renal insufficiency and chronic kidney disease have also been reported to be an independent risk factor for POP after cardiac surgery in previous literature (7, 19). Kinlin et al. found that patients with preoperative creatinine levels >1.2 mg/dL had a 1.6-fold increased risk of POP after CABG (7). Hortal et al. found that patients with preoperative creatinine levels >1.5 mg/dL had a 2.2-fold increased risk of POP after cardiac surgery (28). A series of studies by Wang et al. showed that patients with renal insufficiency had a 2.7-fold increased risk of POP after cardiac surgery, a 2.1-fold increased risk of POP after elective cardiac surgery, a 2.7-fold increased risk of POP after valve surgery, a 2.5-fold increased risk of POP after aortic dissection surgery, and the risk of POP increased with creatinine levels after redo cardiac surgery (8, 9, 19, 20, 26). Therefore, preoperative improvement of renal function may be a feasible strategy to reduce complications and improve prognosis in patients with renal dysfunction.

#### Cardiac Surgery History

Patients with a history of cardiac surgery have a significantly increased risk of postoperative complications such as POP and in-hospital mortality, which has been confirmed in multiple studies (9, 19, 20, 26, 38). Compared with patients undergoing primary surgery, patients undergoing redo cardiac surgery tend to have poorer physical fitness and tolerance, more underlying diseases, more complicated anatomy and surgical operations. Therefore, the risk of postoperative adverse outcomes and death tends to be higher in this surgical population (38, 39). Hortal et al. found that the risk of POP increased 3 times in patients with cardiac surgery history (28). Kinlin et al. found that the risk of POP after CABG was 2.3 times higher in patients with previous internal mammary artery transplantation (7). Bianco et al. compared the underlying disease conditions between primary and redo cardiac surgery patients through a large propensity-matched cohort, finding that patients with cardiac surgery history had more comorbidities, more blood transfusion, longer mechanical ventilation, and higher postoperative mortality (39). Similar results were also obtained by Norton et al. who found that compared with primary surgery patients, patients undergoing redo cardiac surgery had older age, more underlying diseases, more complicated procedures, more intraoperative blood transfusion, and longer cardiopulmonary bypass time (40).

#### Cardiac Function

Poor cardiac function as an independent risk factor for POP after cardiac surgery has been demonstrated in numerous previous studies (5, 10, 24, 28, 41–43). Strobel et al. found that left ventricular ejection fraction was inversely associated with the risk of POP after CABG, which meant that the lower the left ventricular ejection fraction, the higher the risk of POP (10). In patients requiring intra-aortic balloon pumping, the risk of POP increased to 1.59 times. Hortal et al. found that the need for intraoperative inotropic support was identified as an independent risk factor for POP after cardiac surgery in the multivariate regression analysis (28). The risk of POP increased 2.2 times in patients with NYHA class IV, which was included as a predictor into their risk scoring model. Similar results were also obtained by Kilic et al. (24). In their study, the need for preoperative and intraoperative intra-aortic balloon pumping was identified as an independent risk factor and was included in their final risk prediction model, which was associated with a 2.01-fold increased risk of POP after cardiac surgery.

#### Anemia

Preoperative anemia, low red blood cell count, low hemoglobin levels and low hematocrit have also been reported to be closely associated with the development of POP after cardiac surgery in previous literature (11, 12, 27, 44). Ailawadi et al. found that hemoglobin levels were inversely associated with the risk of POP after cardiac surgery and lower hemoglobin level was identified as an independent risk factor in the multivariate regression analysis (27). Similar results were also obtained by Strobel et al. who found that hematocrit was inversely associated with the risk of POP after CABG, and lower hematocrit was identified as a predictor for POP in their analysis (10). In a study by Wang et al. preoperative anemia was identified as an independent risk factor in multivariate regression analysis, with a 1.6-fold increased risk of POP compared with patients without that (20). Canet et al. obtained similar results in a cohort study, and the risk of POP reached 3 times higher in this population (45). As the largest amount of blood cells in human body, the main function of red blood cells is transporting oxygen and carbon dioxide, participating in acid-base balance regulation, and modulating the clearance of circulating immune complexes. Therefore, attention should be paid to strengthening nutrition, enhancing immunity and improving anemia for patients with preoperative anemia. In recent years, the research on the effect of preoperative hematocrit on blood transfusion has gradually increased, but the optimal threshold for transfusion remains to be further investigated (46). Meanwhile, additional attention should be paid to reducing intraoperative bleeding and close observation should be given to prevent the development of POP and other adverse outcomes (45).

#### Body Mass Index

BMI has been reported to be an independent predictor for POP after cardiovascular surgery in several previous studies (7, 20, 43). Xiao et al. found that the BMI of patients with POP after mitral valve replacement was significantly higher compared with patients without POP, and higher BMI was identified as an independent predictor for POP in the multivariate analysis (43). Wang et al. divided BMI into two groups based on a 24 kg/m^2^ boundary and found that BMI ≥ 24 kg/m^2^ was an independent risk factor for POP after cardiac surgery (20). However, Kinlin et al. had different findings in their study (7). They found that lower body weight was independently associated with the development of POP after cardiac surgery, with a 2.89-fold increased risk. In the results of Santos et al. the risk reached up to 9.8 times in patients with lower body weight (44). These findings suggest that whether overweight and obesity or underweight and wasting all increase the risk of postoperative complications such as POP after cardiac surgery. Thus, scientific and reasonable diet and proper exercise should be valued to maintain a fine body weight and shape and resultant health. In addition, a positive association between obesity and frailty has been found in previous studies, which was associated with higher risk of morbidity and mortality (47, 48). Therefore, preoperative assessment of frailty index in patients undergoing elective cardiac surgery may be instructive to some extent.

#### Diabetes Mellitus

Diabetes mellitus has also been reported to be a risk factor for POP after cardiovascular surgery in some previous reports (10). Strobel et al. found that patients with diabetes mellitus had a 1.3-fold increased risk of POP after CABG (10). Similar results were also obtained by Wang et al. who found that the risk of POP after cardiac surgery increased to 1.4 times in patients with diabetes mellitus, and the risk increased to 2.1 times in patients undergoing heart valve surgery (9, 20). However, a systematic review and meta-analysis conducted by He et al. found that the association between diabetes mellitus and POP after cardiac surgery was not significant (12). The influence of preoperative level of glycosylated hemoglobin A1c and postoperative glycemic variability on major adverse outcomes following cardiac surgery has been reported in previous studies, which indicated that both had a predictive value for postoperative complications including POP (49–52). Therefore, good glycemic control for patients undergoing elective cardiac surgery may significantly reduce the risk of POP and other adverse outcomes.

#### Other Risk Factors

In addition to those widely reported risk factors mentioned above, some of the following factors have also been reported to be associated with the development of POP after cardiovascular surgery in the literature, including hypertension, cerebrovascular disease, peripheral vascular disease, white blood cell count, platelet count, serum albumin levels, liver disease, pulmonary hypertension, steroid use, and re-intervention (10–12, 20, 21, 24, 27, 42, 53). The presence of these factors may increase the risk of POP and other adverse outcomes to some extent. Therefore, more attention should be paid to reducing the risk of postoperative adverse outcomes and improving prognosis in clinical practice.

### Extrinsic Factors

#### Surgical Types

Cardiovascular surgery as a whole contains numerous sub-types. The risk of various postoperative complications, the lengths of hospitalization and the risk of postoperative death may vary greatly among different surgical types due to different operation procedures, surgical time and complexity of different diseases (8, 9). According to clinical experience and previous reports, the risk of POP after multiple types of combined cardiovascular surgery is higher than that of single type of cardiovascular surgery. Wang et al. found that compared with patients undergoing isolated heart valve surgery, the risk of POP increased 3.2 times in patients undergoing combined CABG, 2.9 times in patients undergoing combined aortic surgery, and 4.8 times in patients undergoing combined coronary and aortic surgery (9). For isolated type of cardiovascular surgery, aortic dissection surgery has a much higher risk of POP and other postoperative complications than other surgical types due to its more complicated operation, greater trauma and longer operation time (8). In the findings of Hortal et al. ascending aorta surgery was an independent risk factor for POP, with a 6.2-fold increased risk compared with other types of cardiovascular surgery (53). In the analysis of Wang et al. the probability of POP after Stanford type A aortic dissection was 34.6%, and the rate reached up to 36.9% in the study of Yao et al. (8, 21). Meanwhile, compared with elective cardiac surgery, patients undergoing emergency surgery may have a significantly increased risk of POP (28, 53). In the findings of Strobel et al. patients undergoing emergency surgery had a 2.2-fold increased risk of POP after CABG (10). In addition, patients undergoing surgeries for end stage heart failure such as left ventricular assist device insertion and heart transplantation had ultrahigh risk of developing POP (54–56). A single-center retrospective study conducted by Pons et al. reported that pneumonia was the most common infectious complications postoperatively, which developed in 52.5% of the patients undergoing heart transplantation (57).

#### Cardiopulmonary Bypass Time

CPB time has been widely reported to be associated with the development of POP after cardiovascular surgery in previous studies (12, 19, 24, 58). In a meta-analysis and systematic review, He et al. concluded that prolonged duration of CPB was significantly associated with an increased risk of POP (12). In the results of Olga et al. CPB time was positively associated with the risk of POP after cardiac surgery, and the risk increased by 1% for each additional minute (58). Kilic et al. found that patients on CPB for more than 100 min had a 1.7-fold increased risk nt of various complications and aof POP after cardiac surgery (24). Allou et al. also reported a positive relationship between CPB duration and POP after cardiac surgery, and the risk of POP increased significantly with the extension of CPB duration in their multivariate analysis (41). After selection and inclusion of interaction between variables, the interaction between CPB time >60 min and intraoperative red blood cell transfusion was identified as an independent predictor and included in the final risk model, and the risk of POP increased to 2.98 times in these patients.

CPB can reduce lung compliance and lead to pulmonary dysfunction by inducing ischemia-reperfusion injury and systemic inflammatory response (59). However, the importance of CPB in the developmedverse outcomes is often underestimated or even ignored by some cardiac surgeons. In recent years, minimally invasive CPB (MICPB) has been introduced clinically, and the use of MICPB has been reported to significantly improve clinical outcomes (60). MICPB is a closed system that can provide optimal intraoperative perfusion, with near-normal systemic vascular resistance and higher mean arterial pressure, which may reduce the need for vasoactive drugs. In the past few years, this system has developed into the best perfusion technique in clinical practice, however, in the field of science and technology, many trials, improvement and research work are still being conducted to further improve the biocompatibility of MICPB systems and reduce adverse effects (61). Current clinical evidence has demonstrated that MICPB is superior to conventional CPB in reducing hemodilution and better preserving hematocrit, which may reduce the need for perioperative blood transfusion. In addition, this system can significantly attenuate systemic inflammatory response and protect end-organ function (61). A large meta-analysis and systematic review of randomized controlled trials conducted by Anastasiadis et al. showed that the use of MICPB can significantly reduce the morbidity of various complications and mortality after cardiac surgery compared with conventional CPB (62). Recently, they have also introduced the concept of a “more physiologic” cardiac surgery to emphasize the need of further improvement of patient outcomes (63).

#### The Amount of Blood Transfusion

The amount of blood transfusion has also been widely reported to be an independent risk factor for POP in patients undergoing cardiovascular surgery (19). Although the transfusion of blood and blood products is an important guarantee for the success of the operation and can be lifesaving in cardiovascular surgery, there is increasing evidence that massive blood transfusion may significantly increase the risk of adverse outcomes (64–67). Likosky et al. conducted a large prospective multicenter cohort study to explore the relationship between blood transfusion and POP in patients undergoing CABG, finding that patients who received blood transfusion had a 3.4-fold increased risk of POP, and the risk increased significantly with each additional unit of red blood cells transfused, showing a dose-effect relationship (68). The results of Wang et al. showed that patients who were intraoperatively transfused had a 3.5-fold increased risk of POP after general cardiac surgery, a 2.8-fold increased risk in valve surgery, and the risk increased by 23.2% with each additional unit of red blood cells transfused in aortic dissection surgery, showing a dose-effect relationship (8, 9, 20). However, the results of a multicenter study conducted by Lapar et al. showed that limiting the amount of perioperative blood transfusion can not only reduce the risk of mortality and postoperative complications such as POP, but also greatly reduce the medical burden (69). Therefore, in clinical practice, unnecessary blood transfusion should be avoided, blood transfusion indications should be strictly controlled, and autologous blood transfusion can be used when conditions permit, so as to reduce the risk of POP and other blood transfusion-related diseases (70, 71). At the same time, proper fluid supplementation before operation, rapid and precise operation during operation, improvement of surgical techniques and hemostasis means, and shortening of operation time may be helpful for effective control of bleeding and resultant less need for transfusion (24).

The association between allogeneic blood transfusion and increased risk of POP can be partly explained by changes in immune function (72, 73). In addition, longer storage time of blood and blood products has also been reported to be associated with an increased risk of POP and worse prognosis, which may be due to the reduced oxygen-carrying capacity of red blood cells and transfusion-related inflammatory responses (74–76). Although massive blood transfusion is often due to massive blood loss and anemia in clinical practice, according to the recommendations of clinical practice guidelines, restrictive blood transfusion strategies are still strongly recommended to reduce the risk of postoperative complications and improve prognosis, even in major operations such as cardiovascular surgery (77–79). In addition, the exploration of blood transfusion replacement therapy and autologous blood transfusion may be new research directions in future work (80).

#### Duration of Mechanical Ventilation

The use of ventilator and mechanical ventilation are indispensable life support techniques for cardiovascular surgery. However, prolonged mechanical ventilation has been reported to be strongly associated with an increased risk of POP in many studies, which is associated with the damage to the defense mechanism of the respiratory system caused by tracheal intubation (5, 42, 53, 81, 82). The use of endotracheal intubation and ventilator changes the normal airway structure and damages the natural barrier of the respiratory system, resulting in a weakened cough reflex, disappearance of the gag reflex, weakened ciliary movement, reduced ability to clear secretions, and damage to the defenses of the upper respiratory tract. These changes make the pathogens in the upper respiratory tract such as the oral cavity and throat more likely to migrate to the lower respiratory tract, and then colonize and breed in the distal bronchi and lung tissue, thereby leading to the development of POP. It has been reported that the risk of POP increases by 1–3% for each additional day of mechanical ventilation, and endotracheal intubation should be removed as soon as possible when the extubation conditions are met (83, 84). In addition, prolonged mechanical ventilation can cause diaphragmatic atrophy, which may significantly increase the risk of POP and affect prognosis (85).

Hortal et al. found that the duration of mechanical ventilation was an independent predictor for POP after cardiac surgery in the multivariate analysis, and the risk increased significantly with the extension of mechanical ventilation, showing a dose-effect relationship (28). Bouza et al. found that patients who were mechanically ventilated for more than 96 h had a 12.3-fold increased risk of POP after cardiac surgery, and the in-hospital mortality increased significantly in these patients (42). In a meta-analysis and systematic review conducted by He et al. the overall incidence of POP after cardiac surgery was 6.37%, however, the incidence reach 35.2% in patients mechanically ventilated for more than 48 h, which demonstrated that prolonged mechanical ventilation was significantly associated with an increased risk of POP (12). Therefore, close attention should be paid to the recovery of respiratory function and more stringent ventilation strategies should be implemented, so as to shorten the duration of mechanical ventilation, reduce the risk of postoperative complications, and improve prognosis.

#### Repeated Endotracheal Intubation

Repeated tracheal intubation has also been identified as an independent risk factor for POP after cardiovascular surgery in the literature (86–90). Tracheal intubation may inevitably destroy the integrity of the airway mucosa, and the operation of reintubation can cause more damage to the respiratory tract, thus can significantly increase the risk of POP. Sheng et al. found that reintubation was an independent predictor for POP after cardiac surgery, with an 8.9-fold increased risk in patients who were reintubated compared with patients without reintubation (81). In the results of Hortal et al. the risk of POP was as high as 14.3 times in patients reintubated (28). In a systematic review and meta-analysis by Gao et al. they found that reintubation significantly increased the risk of POP and in-hospital mortality, and the risk of extubation failure after cardiac surgery increased significantly compared with other surgical types (89). He et al. obtained similar results and concluded that reintubation was significantly associated with the development of POP after cardiac surgery (12). Garcia et al. found that appropriate application of non-invasive ventilation after extubation could avoid the respiratory tract injury caused by reintubation and consequent increased risk of POP and death, however, non-invasive ventilation should be performed under strict conditions and standards (91).

## Interventions

### Preoperative Respiratory Physiotherapy

Previous studies have shown that preoperative respiratory physiotherapy can significantly reduce the incidence of postoperative complications such as POP and atelectasis (13, 37, 92). In order to explore the preventive effect of short-term preoperative inspiratory muscle training on the incidence of postoperative pulmonary complications in patients undergoing elective cardiac surgery, Chen et al. conducted a randomized controlled trial in which the intervention group received 5 days of preoperative respiratory muscle training on the basis of routine care (15). The results showed that compared to the control group, patients in the intervention group had significantly reduced incidence of pulmonary complications, shortened postoperative hospital stay, and a significant increase in inspiratory muscle strength, forced expiratory volume in the first second of expiration, forced vital capacity and maximal voluntary ventilation. In a systematic review and meta-analysis conducted by Neto et al. they found that preoperative inspiratory muscle training could result in improvement in the inspiratory muscle strength, endurance, forced expiratory volume in 1 second, forced vital capacity, duration of hospital stay and reduced risk of postoperative pulmonary complications, and postoperative inspiratory muscle training could result in significant improvement in tidal volume, maximal inspiratory pressure and peak expiratory flow in patients undergoing cardiac surgery (93). Another systematic review and meta-analysis conducted by Kendall et al. showed that inspiratory muscle training was effective to reduce postoperative pulmonary complications and length of hospital stay (94). Similar results were also obtained by Thybo et al. who found that preoperative inspiratory muscle training may significantly reduce the risk of developing POP and atelectasis in patients undergoing CABG and heart valve surgery (95).

Turquetto et al. conducted a randomized controlled trial studying the effects of aerobic exercise and inspiratory muscle training on functional capacity, pulmonary function and autonomic control in patients after Fontan operation, finding that inspiratory muscle training cound significantly improve functional capacity and increase inspiratory muscle strength and spirometry (13). Another similar randomized controlled trial conducted by Cordeiro et al. showed that inspiratory muscle training was effective in improving functional capacity and inspiratory muscle strength in patients undergoing cardiac surgery (96). Santos et al. found that short-term moderate-to-high intensity inspiratory muscle training plus aerobic and resistance exercise could provide additional benefits in exercise capacity, inspiratory muscle strength, inspiratory muscle endurance, 6-min walk test, quality of life, and antioxidant profile in patients undergoing CABG (97). Despite differences in the specific measures and methods adopted across the studies, previous findings consistently suggested that preoperative respiratory physiotherapy could significantly improve patient outcomes. Therefore, it may be a feasible strategy to reduce postoperative respiratory complications and improve outcomes to implement appropriate respiratory physiotherapy in patients undergoing elective cardiac surgery in clinical practice.

### Oral Care

A growing number of studies have found that active and systematic preoperative oral care can play an important role in preventing and reducing the development of POP after cardiovascular surgery, among which tooth brushing is one of the most acceptable and common measures. Oral care intervention by multiple tooth brushing can effectively remove oropharyngeal pathogens, improve oral hygiene, and reduce the risk of POP (98). In addition to tooth brushing, the commonly used oral care methods are mainly the use of chlorhexidine gluconate mouthwash and the combination of the two.

A prospective study conducted by Nicolosi et al. found that tooth brushing plus 0.12% chlorhexidine gluconate oral rinse was effective in preventing ventilator-associated pneumonia after cardiovascular surgery (99). The risk of POP was 3-fold higher and the hospital stay was significantly prolonged in patients without preoperative oral care. A systematic review and meta-analysis conducted by Bardia et al. demonstrated that the use of chlorhexidine gluconate was associated with reduced risk of POP after cardiac surgery, and this protective effect was particularly profound for gram-positive organisms (100). Among the patients receiving preoperative chlorhexidine mouthwash, the risk of POP was reduced by approximately one-half and the outcomes could be significantly improved. In addition, None of these studies reported adverse effects of chlorhexidine gluconate mouthwash.

To investigate the potential cost-effectiveness of administering preoperative chlorhexidine mouthwash at reducing POP among patients undergoing abdominal surgery, Kachapila et al. constructed a decision analytic model to compare costs and benefits within the first 30 days postoperatively (101). They found that patients receiving mouthwash had lower average costs and lower proportion of POP, and mouthwash surgery was more likely to be cost-effective. To explore the association between perioperative chlorhexidine oral care and POP in non-cardiac surgical patients, Liang et al. conducted a systematic review and meta-analysis (102). They found that nurse-led chlorhexidine oral care could significantly decrease the risk of POP and could be more convenient and economical than dental professional-led perioperative oral care.

### Subglottic Secretion Drainage

Subglottic secretion drainage refers to a technique of continuous or intermittent suction during mechanical ventilation in order to remove secretions and retention from the subglottis and air sacs. The subglottic secretions contain a large number of pathogens, which is an important source of pathogenic bacteria for POP after cardiovascular surgery. Previous studies have shown that subglottic secretion drainage is of great value for the prevention of POP after cardiovascular surgery. Hudson et al. conducted a large retrospective observational study to explore the impact of subglottic suction on the incidence of POP after cardiac surgery (16). They found that continuous aspiration of subglottic secretions could significantly reduce the risk of POP and 30-day in-hospital mortality and shorten the lengths of mechanical ventilation and ICU stay. Similar results were also obtained by Granda et al. who found that the use of aspiration of subglottic secretions in patients undergoing cardiac surgery could significantly reduce the incidence density of POP, days on mechanical ventilation, the use of antibiotics, and medical burden (103). They considered this to be a very effective procedure and recommended that all patients undergoing major heart surgery should routinely receive aspiration of subglottic secretions from the moment of induction of anesthesia rather than entering ICU after surgery. Another randomized controlled trial conducted by Bouza et al. also showed that the use of continuous aspiration of subglottic secretions in patients undergoing major heart surgery could significantly reduce the risk of POP, shorten the length of hospital stay, reduce the amount of antibiotics and medical costs (88). Therefore, implementing timely and regular subglottic suction to remove and prevent secretions from flowing into the lower respiratory tract is an important preventive measure for POP in patients undergoing cardiovascular surgery.

### Other Interventions

Some other measures have also been reported to be effective in prevention and treatment, including lung protective ventilation management, alveolar recruitment maneuvers, early mobilization and progressive ambulation, elevation of the head end and semi-recumbent positioning, sedation vacation and the use of a weaning protocol, early extubation, strict changes of ventilatory circuit tube, appropriate cuff pressure control, use of non-invasive ventilation, proper use of tracheostomy, enteral feeding, selective digestive decontamination, peptic ulcer disease prophylaxis, silver-coated endotracheal tubes, microbiological surveillance, and deep vein thrombosis prophylaxis (104–109). In recent years, some prevention care bundles and prevention checklists which consist a series of rationalized measures have been developed and validated to be effective and convenient in preventing POP and shortening the duration of mechanical ventilation. The combined use of several interventions is currently recommended in clinical practice (109, 110). For high-risk patients, it may be effective to reduce the risk of POP and improve clinical outcomes by implementing multiple interventions such as respiratory physiotherapy, oral care, subglottic secretion drainage, and other appropriate measures.

In conclusion, POP is one of the most common respiratory complications in patients undergoing cardiovascular surgery, which is closely related to the increase of adverse outcomes and medical burden. Through the review of the current status, risk factors and intervention measures, this article provides new ideas for clinical prevention and treatment of POP after cardiovascular surgery.

## Author Contributions

FX, FS, and DW: conception, design, and writing. XH, YL, and XD: administrative support. ZJ, MS, DW, and FX: collection, assembly, and analysis of data. All authors read and approved the final manuscript.

## Funding

This work was supported by a grant from the National Natural Science Foundation of China (No. 81800413).

## Conflict of Interest

The authors declare that the research was conducted in the absence of any commercial or financial relationships that could be construed as a potential conflict of interest.

## Publisher's Note

All claims expressed in this article are solely those of the authors and do not necessarily represent those of their affiliated organizations, or those of the publisher, the editors and the reviewers. Any product that may be evaluated in this article, or claim that may be made by its manufacturer, is not guaranteed or endorsed by the publisher.

## Abbreviations

BMI: body mass index
CABG: coronary artery bypass grafting
CPB: cardiopulmonary bypass
ICU: intensive care unit
POP: postoperative pneumonia.
